# Effects of rhythmic visual cues on cortical activation and functional connectivity features during stepping: an fNIRS study

**DOI:** 10.3389/fnhum.2024.1337504

**Published:** 2024-02-12

**Authors:** Jiajia Wu, Huilin Zhou, Hao Chen, Wensong Jiang, Xuelian Wang, Tao Meng, Chaowen Wu, Li Li, Yuemin Wu, Weinv Fan, Changcheng Shi, Guokun Zuo

**Affiliations:** ^1^Cixi Biomedical Research Institute, Wenzhou Medical University, Ningbo, Zhejiang, China; ^2^Ningbo Institute of Materials Technology and Engineering, Chinese Academy of Sciences, Ningbo, Zhejiang, China; ^3^Ningbo Cixi Institute of Biomedical Engineering, Ningbo, Zhejiang, China; ^4^Department of Neurology, Ningbo No. 2 Hospital, Ningbo, Zhejiang, China

**Keywords:** rhythmic visual cues, stepping, functional near-infrared spectroscopy, cortical activity, functional connectivity

## Abstract

**Introduction:**

Rhythmic visual cues (RVCs) may influence gait initiation by modulating cognition resources. However, it is unknown how RVCs modulate cognitive resources allocation during gait movements. This study focused on investigating the effects of RVCs on cortical hemodynamic response features during stepping to evaluate the changes of cognitive resources.

**Methods:**

We recorded cerebral hemoglobin concentration changes of 14 channels in 17 healthy subjects using functional near-infrared spectroscopy (fNIRS) during stepping tasks under exposure to RVCs and non-rhythmic visual cues (NRVCs). We reported mean oxygenated hemoglobin (HbO) concentration changes, β-values, and functional connectivity (FC) between channels.

**Results:**

The results showed that, the RVC conditions revealed lower HbO responses compared to the NRVC conditions during the preparation and early stepping. Correspondingly, the β-values reflected that RVCs elicited lower hemodynamic responses than NRVCs, and there was a decreasing trend in stimulus-evoked cortical activation as the task progressed. However, the FC between channels were stronger under RVCs than under NRVCs during the stepping progress, and there were more significant differences in FC during the early stepping.

**Discussion:**

In conclusion, there were lower cognitive demand and stronger FC under RVC conditions than NRVC conditions, which indicated higher efficiency of cognitive resources allocation during stepping tasks. This study may provide a new insight for further understanding the mechanism on how RVCs alleviate freezing of gait.

## 1 Introduction

Healthy and safe walking is considered to be associated with cognitive abilities (Morris et al., 2017). For people with Parkinson’s disease (PD), their ability to walk safely in daily environments has been impaired, with freezing of gait (FoG) being one of the most severe gait dysfunctions in PD. Recent studies have shown that external stimuli such as auditory, visual, and tactile cues have been used as effective ways to reduce FoG symptoms in patients with PD (Keus et al., 2007; Lee et al., 2012). In particular, a number of studies have shown that rhythmic cues can modulate and improve FoG symptoms and have been proven by many researchers to be effective treatments for recovering gait function in PD patients with FoG (PD-FoG) (Lim et al., 2005; Nombela et al., 2013; Spaulding et al., 2013; Lu et al., 2017). Researchers have compared various rhythmic cues and found that rhythmic visual cues (RVCs) (e.g., interval lines on the floor) worked better (Lee et al., 2012; McCandless et al., 2016).

However, to our knowledge, few studies have been conducted on brain activity during gait movement under RVCs. In a study that generated a visual cue in the form of checkerboard tiles, the beta band information flow in the visual cortex and sensorimotor cortex was increased during gait movement in response to the visual cue (Velu et al., 2014). In addition, an electroencephalography (EEG) study revealed reduced alpha and beta band energy in the sensory cortex of PD patients while walking on a ground with transverse black tape lines, which may be related to their need for increased visual attention processing and the use of additional visual information to complete tasks (Stuart et al., 2021). In a previous EEG study, we initially explored and found lower contingent negative variance (CNV) amplitudes during gait initiation preparation in the RVC conditions compared to the non-rhythmic visual cue (NRVC) conditions (Zhou et al., 2021), and the CNV amplitude is considered to be related to the level of cognitive effort and the allocation of cognitive resources (Ansari and Derakshan, 2011). Therefore, the results indicated that RVCs may reduce the cognitive resources demand that triggers gait initiation. And in another article, we have analyzed the brain functional network during gait initiation which showed stronger interactions between brain regions in the RVC conditions (Yan et al., 2023). But it remained unclear whether RVCs have the same effect on the allocation of cognitive resources during continuous movement processes (e.g., walking).

Recent technological developments in neuroimaging allow the recording of brain activity during real-time gait. Functional near-infrared spectroscopy (fNIRS), a non-invasive optical imaging technique, allows the recording of changes in blood oxygen concentration in the cerebral cortex, with the advantages of portability and resistance to motion artifacts. A number of studies have explored cortical activity during walking using fNIRS (Koenraadt et al., 2014; Maidan et al., 2016; Stuart et al., 2019), but there have been no studies on the differences in cortical blood oxygen changes during gait with and without RVCs. Our previous EEG studies focused only on the process of gait initiation and preparation, (Zhou et al., 2021; Yan et al., 2023), but did not explore the effect of RVCs on brain activity during sustained gait movements after gait initiation. Since data recorded by EEG are susceptible to artifacts related to muscle and head movements, fNIRS remains a well-suited option.

Most fNIRS studies of brain activity changes during continuous walking or turning in PD patients have focused on the prefrontal cortex (PFC) (Maidan et al., 2015; Belluscio et al., 2019; Stuart et al., 2019), and these studies have proposed that additional activation of the PFC is also a contributing factor to FoG episodes. Correlative studies in neuroimaging have shown that the perception of rhythm activates key motor networks that are impaired in PD patients, such as premotor and supplementary motor areas (Nombela et al., 2013). Several fNIRS studies have confirmed that prefrontal cortex, primary sensorimotor areas and supplementary motor areas play an important role in gait control (Harada et al., 2009; Kurz et al., 2012; Koenraadt et al., 2014). The cerebral cortex is the most advanced and complex central area of motor regulation and relies on interactions between cortices for its function. To understand the cognitive functions of brain, it is important to understand how the brain balances local processing and global integration (Dragomir and Omurtag, 2020). Functional connectivity (FC) can reflect the functional synchronization between different spatial regions of the cerebral cortex and the degree of coordination and cooperation. Sasai et al. (2012) simultaneously used fNIRS and functional magnetic resonance imaging (fMRI) to conduct resting-state functional connectivity analysis and found that the results of the two were highly consistent, which proved the feasibility of using fNIRS to conduct resting-state functional connectivity research. A functional connectivity study based on fNIRS revealed the cognitive resource allocation and neuroregulatory mechanisms of the aging brain under complex walking conditions by analyzing the activation of the PFC and the functional connectivity between various brain regions in the PFC during dual-task obstacle crossing in the elderly (Chen et al., 2022). For the present study, what fNIRS captured were changes in hemodynamic activity related to local neural activities, while the overall pattern of collaboration between all localities was unclear. Therefore, to further explore the mechanism of brain allocation of cognitive resources, it becomes necessary to analyze the functional connectivity between different channels or brain regions.

The purpose of this study was to investigate the effects of RVCs on cognitive resource allocation during continuous stepping. Therefore, we recorded fNIRS data during stepping tasks in 17 healthy subjects exposed to NRVCs and RVCs. We calculated the changes of oxyhemoglobin (HbO) concentration and cortical activation in the PFC, motor cortices (MC), and somatosensory association cortex (SSAC), and we also calculated the FC between channels. We hypothesized that in the stepping processes, the RVC conditions elicited lower HbO responses and cortical activation than the NRVC conditions. We also hypothesized that the functional connectivity between channels was greater under RVC conditions than under NRVC conditions. From these, we further hypothesized that cognitive resources during stepping would be more efficiently allocated and utilized under RVC conditions compared to NRVC conditions.

## 2 Material and methods

### 2.1 Participants

Seventeen healthy subjects with normal or correct-to-normal vision participated in this experiment. All participants completed the Waterloo Footedness Questionnaire-Revised (WFQ-R) (Elias and Bryden, 1998), and all of them showed right-footed. All of them did not have a history of neurological diseases or orthopedic disorders in the legs. One participant’s data were excluded due to excessive motion artifacts. As a result, data from the remaining 16 participants (mean age 23.8 ± 1.0 years; 5 females and 11 males) were used for the full analysis. This study adhered to the guidelines of the Declaration of Helsinki, and all participants signed a consent form approved by the local Research Ethics Committee before participating in the experimental protocol.

### 2.2 Data acquisition

A continuous wave fNIRS system (LABNIRS; Shimadzu, Kyoto, Japan) with continuous wave laser diodes with wavelengths of 780 ± 5 nm, 805 ± 5 nm and 830 ± 5 nm was utilized to measure changes in the concentrations of oxygenated hemoglobin (HbO), deoxygenated hemoglobin (HbR), and total hemoglobin (HbT). The sampling rate was 42 Hz. Considering the measurement requirements and the number of optodes, we designed a unique 14-channels array consisting of 7 infrared optode emitters and 8 optode detectors that were separated by 30 mm.

**FIGURE 1:**
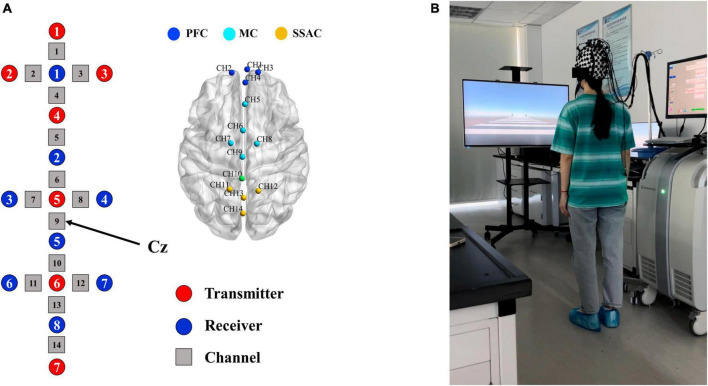
Schematic for arrangement of optodes and experimental setup. **(A)** Layout of the optical optodes and division of ROIs. Red circles represent transmitters, blue circles represent detectors, and gray squares are channels. All channels covered by optodes (except channel 10) were labeled with different colored nodes and divided into three regions of interests (ROIs). **(B)** Experimental setup. The fNIRS optodes cap was placed on the subject’s head.

The fNIRS optodes cap was put on the subject’s head and tightened at the chin to hold it in place (Figure 1B), and the optical optodes arrangement is shown in Figure 1A. Three-dimensional (3D) positions of the emitters and detectors were obtained using a 3D digitizer (FASTRAK, Polhemus, Vermont, USA) in reference to the nasion, central zero (Cz, respect to the International 10–20 system) and bilateral external auditory meatus.

### 2.3 Experimental procedure

**FIGURE 2:**
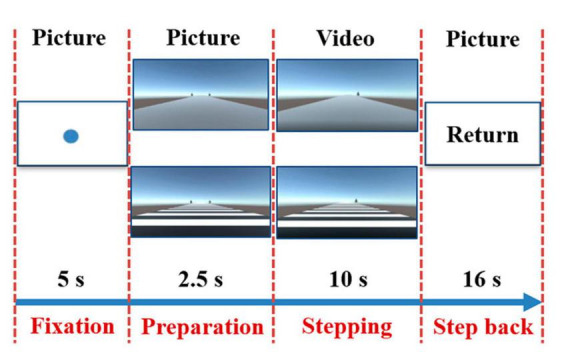
Experimental paradigm. Each trail consisted of four periods. In the first period, a solid blue circle fixation appeared in the center of the screen for 5 s, prompting subjects to maintain a standing position. In the second period, a stationary picture with rhythmic or non-rhythmic cue was presented for 2.5 s to remind subjects to prepare for stepping. In the third period, a dynamic video with the same type of visual cue appeared on the screen for 10 s, prompting subjects to initiate gait at the moment of the occurrence of the dynamic visual cue and subsequently complete stepping tasks. In the final period, a return command appeared for 16 s and reminded the subjects took a step back to return to their original position and stood still for rest.

All subjects completed the stepping task in both the RVCs condition and the NRVCs condition. The scenarios for both the RVCs and the NRVCs were designed via Unity Personal (Unity Technology, San Francisco, USA), as mentioned in our previous study (Zhou et al., 2021). Among them, plain gray pavement was used to represent NRVC and black-and-white stripe pavement was used to represent RVC. In the visual cue scene, we also set up trees on both sides of the road to be used as simulated dynamic walking reference. We then designed the experimental paradigm in E-Prime 3.0, which was displayed on a 65-inch screen located 1 meter in front of where the subjects were standing. A total of four blocks were designed in this experiment, including two NRVC blocks and two RVC blocks. All subjects were required to complete four blocks with the NRVC and RVC occurred alternately. The two blocks that treated NRVC as visual cues constituted the NRVC condition for this study, and the two blocks that treated RVC as visual cues constituted the RVC condition. Each block contained eight repetitive trials. As shown in Figure 2, each trial consisted of four periods: fixation, preparation, stepping, step back.

First, a solid blue circle fixation appeared in the center of the screen for 5 s, prompting the subject to maintain a standing position. Then, a stationary picture with rhythmic or non-rhythmic cue was presented for 2.5 s to remind subjects to prepare for stepping. Next, a dynamic video with the same type of visual cue appeared on the screen for 10 s, prompting subjects to initiate gait at the moment of the occurrence of the dynamic visual cue and subsequently complete stepping tasks. Finally, a return command appeared for 16 s and reminded the subjects took a step back to return to their original position and stood still for rest. Notably, the tree on the left side of the path disappeared as the dynamic cue was triggered, indicating that subjects uniformly initiated with their right foot and then proceeded to complete the stepping task. During the experiment, subjects were asked to decrease unnecessary head movements and swallowing, and most light sources in the laboratory were turned off to minimize the influence of ambient light on fNIRS recordings. The specific experimental process was included in the Supplementary Video 1.

### 2.4 Data analysis

#### 2.4.1 Preprocessing and analysis

Prior to preprocessing, we reduced the sampling rate of the fNIRS data to 15 Hz. Then the data were pre-processed using the HOMER2 toolbox (version 2.8) (Huppert et al., 2009) and analyzed by custom MATLAB (MathWorks, Natick, MA, USA) code. Pre-processing procedures specifically included: (1) converting raw data (intensity) to optical density (OD); (2) detecting motion artifacts due to head movements and swallowing, and the parameter in this process were set as tMotion = 0.7 s, tMask = 2.0, STDEVthresh = 10.0, AMPthresh = 5.00; (3) wavelet algorithm was utilized to remove motion artifacts; (4) bandpass filter between 0.01 and 0.08 Hz was used to eliminate physiological noise (such as cardiac pulsation and respiration) and low-frequency drifts; (5) converting OD to concentrations; (6) calculating the block average according to the stimulation conditions over the time range of the trial. We adopted the moment when the static visual stimulus appeared as the moment of zero and used the 5 s before that moment as the baseline phase. The changes in HbO concentration were obtained by subtracting the average of the baseline from each channel.

From the preprocessed fNIRS data, we used HbO signals as the indicator of hemodynamic response for further analysis, since HbO is more sensitive to cerebral blood flow than HbR (Bai et al., 2020). In the subsequent data analysis, we extracted 12.5 s from the onset of the static visual cue to the end of the task, which was divided into three phases: (1) the 2.5 s of static visual cue presentation were considered as pre-task phase (i.e., preparation); (2) 2.5 to 7.5 s after the onset of static visual cue served as the early-task phase (i.e., early stepping); (3) 7.5 to 12.5 s after the onset of static visual cue served as the late-task phase (i.e., late stepping) (Koenraadt et al., 2014). Based on the 3D positions of the transmitters and detectors, the locations of 14 channels in the standard brain space of the Montreal Neurological Institute (MNI) were estimated for each participant (Figure 1A). The optical imaging data was normalized to the standard stereotaxic space of the Montreal Neurological Institute (MNI) brain template using NIRS-statistical parametric mapping (NIRS-SPM) (Ye et al., 2009). The international 10–20 system was used to determine the exact locations of the optodes, with Cz located in channel 9. According to the standard Brodmann brain localization method, the regions covered by the 14 channels including the frontopolar area, dorsolateral prefrontal cortex, frontal eye fields, premotor cortex and supplementary motor areas, and somatosensory association cortex. We divided all channels into three regions of interest (ROIs) based on function: channel 1–4 cover the prefrontal cortex (PFC), channel 5–9 cover the motor cortices (MC) including the premotor cortex and supplementary motor cortex, and channel 11–14 cover the somatosensory association cortex (SSAC) (Figure 1A).

The mean with standard deviation was calculated for all trials in both visual cue conditions for all subjects within each ROI, respectively. The HbO concentration changes were further averaged for each ROI and each trial in the pre-task, early task, and late task phases for both visual cue conditions. We estimated task-evoked hemodynamic responses in each channel for three task phases by NIRS-SPM using a general linear model (GLM). The GLM equation is as follows:

Y=X⁢β+ε

where *X* is the design matrix (which mainly consists of the reference waves corresponding to the two visual cue conditions), *Y* is the matrix of the fNIRS observed signals, β is the vector of model parameters, and ε is the vector of residuals. Given the design matrix *X* and the observed data *Y* the model parameters β were estimated using the least-squares method. The values of the regression parameter β were then used to indicate the extent to which RVCs and NRVCs contribute to the observed signals in the design matrix, i.e., the magnitude of the hemodynamic response induced by each visual cue condition.

Pearson’s correlation coefficients between channels were calculated using preprocessed HbO concentration changes, and the results obtained were used as functional connectivity (FC) values. The formula is as follows:

$$\rho_{X\,Y}=\frac{c\,o\,v\,\left(X,Y\right)}{\sigma_{X}\,\sigma_{Y}}=\frac{E\,\left(X\,Y\right)-E\,\left(X\right)\,E\,\left(Y\right)}{\sqrt{E\,\left(X^{2}\right)-E^{2}\,\left(X\right)}\,\sqrt{E\,\left(Y^{2}\right)-E^{2}\,\left(Y\right)}}$$

where *X* and Y are the time series of HbO concentration changes in the different channels; *cov*(*X*, *Y*) denotes the covariance of X and Y; *E* (*X*) and *E*(*Y*) denote the mean values of X and Y; σ_*X*_ and σ_*Y*_ denote the standard deviations of X and Y; and ρ_*XY*_ is the Pearson’s correlation coefficient, which is commonly expressed as *r*.

In order to yield normally distributed variants, the correlation coefficient was transformed by Fisher transformation, and the formula is as follows:

$$z=\frac{1}{2}\,l\,n\,\frac{1+r}{1-r}$$

#### 2.4.2 Statistical analysis

IBM SPSS (V 26.0) was used for statistical analysis of the data. For HbO concentration changes, a two-way repeated-measures ANOVA with condition (RVCs vs. NRVCs) and ROI (PFC, MC, SSAC) as repeated factors, was conducted at the pre-task, early-task, and late-task phases, respectively. A two-way repeated-measures ANOVA with condition (RVCs vs. NRVCs) and channel (14 channels) as repeated factors, was used to analyze the β-values at each of the three task phases. A two-way repeated-measures ANOVA with condition (RVCs vs. NRVCs) and channel-channel pair (91 channel-channel pairs) as repeated factors, was used to analyze the functional connectivity strength for each of the three task phases. The level of significance was judged by the *p*-value, and *p* < 0.05 was considered statistically significant throughout the analysis.

## 3 Results

### 3.1 HbO concentration changes

**FIGURE 3:**
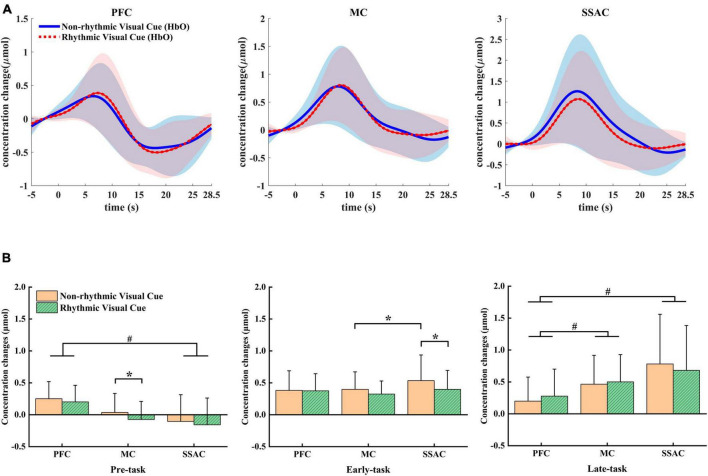
Mean differences in HbO between NRVC and RVC conditions. **(A)** Mean HbO concentration changes across all trials for both conditions within each of the three ROIs. **(B)** Statistical comparison of mean HbO concentration changes between the two conditions within each ROI in each of the three task phases. *p < 0.05, # represents the main effect of ROI.

The grand-averaged HbO concentration changes for all channels within each ROI are shown in Figure 3A along with the standard deviations among all subjects. Moment 0 on the time axis indicated the appearance of a visual cue, and the period from −5 to 0 s was regarded as the baseline. It can be noticed that the HbO concentration began to increase slowly even before the visual cue appeared. In the PFC, HbO responses under the NRVCs were higher than those under the RVCs for a period of approximately 5 s from the appearance of the visual cue. Then, HbO concentrations under the RVCs reached a peak in the middle of the task and began to increase above those under the NRVCs. As the task proceeded, the overall HbO concentration gradually decreased to below the baseline after reaching the peak point and finally returned to the baseline during the resting phase. For MC, the period from the appearance of the visual cue to the middle of the task was characterized by consistently higher HbO level at NRVCs than at RVCs, and after reaching the peak, they both began to decline and gradually approached each other. Differently from PFC and MC, the HbO concentration curve of SSAC under the NRVCs remained above the RVCs throughout the entire process from the appearance of the visual cue until they returned to the baseline levels.

The two-factor repeated measures ANOVA showed that in the pre-task phase, there was a main effect for both condition (*F* = 5.226, *p* = 0.037, η^2^ = 0.258) and ROI (*F* = 10.621, *p* = 0.002, η^2^ = 0.415). In this phase, HbO was always higher under NRVCs than under RVCs, and the HbO responses to PFC were higher than those to both MC and SSAC (Figure 3B). Simple effects analysis for condition showed that MC had significantly higher HbO levels under NRVCs than under RVCs (*p* = 0.018). For ROI, a simple effect analysis found that HbO levels were significantly higher in PFC than in SSAC in either condition (*p* = 0.013). During the early-task phase, the two-way repeated measures ANOVA indicated an interaction effect of condition and ROI (*F* = 5.366, *p* = 0.01, η^2^ = 0.263). *Post-hoc* test analysis showed that during the early-task phase, the HbO concentration of SSAC was significantly higher under NRVCs than under RVCs (*p* = 0.02). The HbO concentration of SSAC under NRVCs was significantly higher than that of MC (*p* = 0.034). For the late-task phase, the main effect of ROI (*F* = 11.318, *p* = 0.001, η^2^ = 0.43) was significant. And there was an interaction effect of condition and ROI (*F* = 7.505, *p* = 0.01, η^2^ = 0.333). Post-hoc test analysis showed that regardless of condition, the HbO concentration of PFC was not only smaller than that of MC (*p* = 0.013), but also smaller than that of SSAC (*p* = 0.004), but there was no significant difference in HbO concentration changes between SSAC and MC.

### 3.2 β-values: cortical activation

**FIGURE 4:**
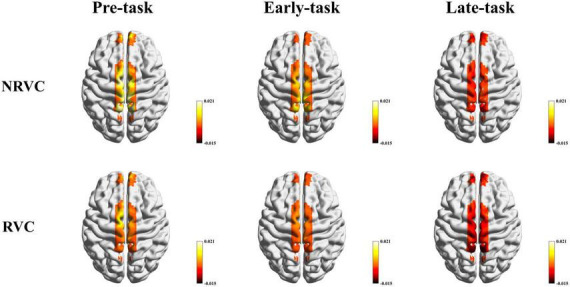
β-based cortical activation maps for the RVC and NRVC conditions in three task phases. Scale indicates the range of beta values.

The β-values can reflect the state of brain activation and can also represent how much different stimulus conditions affect the hemodynamic response. Cortical activation maps using β-values of the RVC and NRVC conditions for each of the three task phases are shown in Figure 4. The activation maps were visualized with the BrainNet Viewer (Xia et al., 2013). The two-factor repeated measurement ANOVA revealed that during the pre-task phase, there was a significant main effect of the condition (*F* = 6.896, *p* = 0.019, η^2^ = 0.315), indicating that cortical activation caused by NRVCs was greater than that caused by RVCs. Simple effect analysis showed that especially the activation of SSAC under NRVCs was significantly higher compared to RVCs (channel 11: *p* = 0.045; channel 12: *p* = 0.017; channel 13: *p* = 0.019). In the early-task phase, there was also a significant main effect of the condition (*F* = 6.006, *p* = 0.027, η^2^ = 0.286). The simple effect of the condition showed that the impact of stimulus conditions on hemodynamic response during this phase decreased compared to the pre-task phase and specifically in channel 8 (*p* = 0.029) and channels 11–13 (channel 11: *p* = 0.033; channel 12: *p* = 0.037; channel 13: *p* = 0.015), where responses were significantly smaller under RVCs than under NRVCs. In the late-task phase, it indicated significant main effects for both condition (*F* = 12.504, *p* = 0.003, η^2^ = 0.455) and channel (*F* = 2.822, *p* = 0.042, η^2^ = 0.158). Further simple effect analysis of condition revealed channels in each ROI with significant differences between two visual cue conditions in the late task-phase (channel 1: *p* = 0.034; channel 8: *p* = 0.008; channel 11: *p* = 0.023; channel 13: *p* = 0.016). Numerically, there was a decreasing trend in β-values over time.

### 3.3 Functional connectivity

**FIGURE 5:**
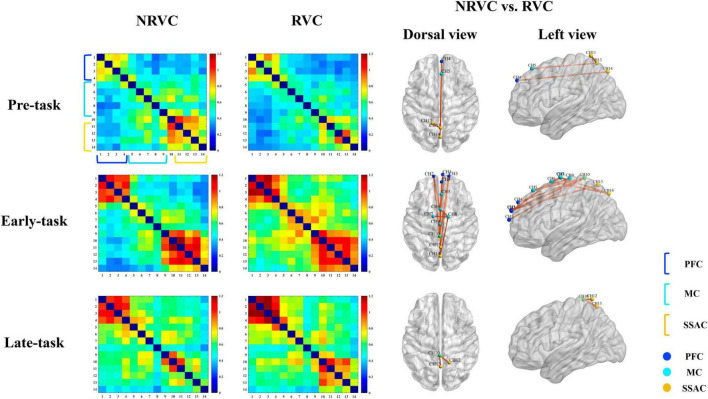
Correlation of functional connectivity between channels in each of the three task phases under RVC and NRVC conditions. Each row from top to bottom corresponds to the results of one task phase, and the first column from left to right is the group-averaged correlation matrix under NRVCs, where each pixel of the matrix indicates the strength of the connection between every two corresponding channel pairs; the second column is the group-averaged correlation matrix under RVCs; and the last two columns are channel-channel pairs whose functional connectivity significantly differed between the two conditions of NRVC versus RVC, where the third column is the dorsal view, the fourth column is the left view. The channels belonging to different ROIs in the correlation matrix and functional connectivity maps are marked with different colors in the legend at the bottom right.

To further investigate the differences in cerebral functional connectivity between the two visual cueing conditions, we calculated the Pearson correlation coefficients between the channel as the functional connectivity (FC). As shown in Figure 5, it contains the correlation coefficient matrices of the channel-channel pairs in the two conditions for each of the three task phases, as well as the FC maps representing the significant differences in FC between the two conditions. We still visualize these functional connection difference with the BrainNet viewer (Xia et al., 2013).

Functional connectivity between the internal channels of the PFC was robust regardless of condition and task phase. Functional connectivity between the internal channels of the SSAC during the early-task phase was also strong. The overall color tone of the correlation coefficient matrix under NRVCs was warmer than that of RVCs during the pre-task phase, while the exact opposite was true for the early-task phase. A two-way repeated measures ANOVA showed that condition (*F* = 3.802, *p* = 0.07, η^2^ = 0.202) did not have a main effect during the early-task phase, but there was a trend toward significance. We did a simple effect analysis for the condition which was of concern, and the results indicated the existence of channel-channel pairs which differed significantly between the two visual cue conditions, as shown in Table 1.

**TABLE 1:** Channel-channel pairs with significant differences in functional connectivity between the two visual cue conditions at three task phases.

Phase	Ch-Ch pair	NRVCs vs. RVCs		
		**Differential mean**	**Standard deviation**	***P*-value**
Pre-task	4–14	0.1514	0.2522	0.030
	5–13	0.0820	0.3163	0.036
	11–13	0.1795	0.3242	0.043
Early-task	1–10	−0.3274	0.5953	0.044
	2–6	−0.2045	0.3273	0.025
	2–10	−0.3008	0.4510	0.018
	3–10	−0.3507	0.4952	0.013
	3–13	−0.2615	0.4747	0.044
	4–10	−0.2707	0.4230	0.022
	5–9	−0.2150	0.3048	0.013
	5–10	−0.2801	0.3826	0.010
	6–8	−0.2558	0.4687	0.045
	6–14	−0.2325	0.4220	0.044
	7–8	−0.2449	0.4110	0.031
	8–14	−0.2051	0.3754	0.045
Late-task	10–12	−0.2478	0.4304	0.036
	10–13	−0.2263	0.4177	0.047

NRVCs, non-rhythmic visual cues; RVCs, rhythmic visual cues; Ch, channel.

## 4 Discussion

The current fNIRS study recorded hemodynamic responses in multiple cortical areas during stepping under RVCs and NRVCs. The findings show that HbO responses under RVCs compared to NRVCs were lower in the pre-task and early task phases, progressively higher in the later task phases, but consistently lower than NRVCs in the SSAC. Functional connectivity between channels was the strongest in the early part of the task. Moreover, the functional connectivity (FC) values were stronger under RVCs than under NRVCs during the stepping progress. The hemodynamic response induced by NRVCs was greater than RVCs and cortical activation declined as the task progressed.

### 4.1 HbO concentration changes

Mean HbO concentration changes in RVCs were lower than in NRVCs during the pre-task and early-task phases in all channels. We divided the channels into three ROIs according to cortical function and averaged the HbO concentration changes of each ROI across the time courses. The PFC plays a crucial role in executive function, particularly in the dorsolateral prefrontal cortex (DLPFC) (Radel et al., 2017), and is inextricably linked to motor planning, attention allocation, and cognitive control (Wood and Grafman, 2003; Panikratova et al., 2020). In the RVC conditions, HbO concentrations were below the NRVC conditions for a period of approximately 5 s after the visual cue appeared. This period consisted of a 2.5 s static visual cue (i.e., the motor preparation phase) and a short period of the early-task. The lower HbO levels in the RVCs may be due to the fact that this visual cue can reduce the cognitive load in the preliminary phase, which was in line with the results of an EEG study in our series (Zhou et al., 2021). In general, the hemodynamic response peaks approximately 5 s after stimulus presentation and returns to baseline approximately 16 s after stimulus onset (Friston et al., 1998). In contrast, the EEG detection technique possesses high temporal resolution, and the signals captured may be neuroelectric activities accurate to a few hundred milliseconds before and after stimulus onset. Based on neurovascular coupling, changes in HbO concentration within the local capillary network of the brain are caused by neuronal firing induced by brain activity (Huppert et al., 2006; Iadecola, 2017). Therefore, it becomes understandable that the lesser cognitive resource demands of the RVCs, as reflected by the motor readiness potentials observed in the EEG research (Zhou et al., 2021), can be mapped to the same conclusions that can be drawn from the blood oxygenation changes on a longer time scale.

For MC, HbO levels were consistently higher in the NRVCs than in the RVCs during the period from the appearance of the visual cue to the middle of the task, reaching a peak after which the two began to decline and approach each other gradually. Prior to peaking, the HbO responses of MC under RVCs were significantly lower than that of NRVCs, which may also be due to the fact that less cognitive resources are required for locomotion under RVCs. The areas covered by MC include the premotor cortex (PMC) and the supplementary motor area (SMA). SMA plays a central role in locomotor control, and it has been claimed that the SMA has also been strongly associated with the initiation of gait in PD-FoG (Brugger et al., 2020). Both the PMC and SMA are involved in motor preparation (Simon et al., 2002; Suzuki et al., 2008), in addition to which the PMC plays a role in the rhythmic generation of externally cued movements and in the organization of the sensorimotor space (Halsband et al., 1993), which may be one of the reasons for the modest increase in the HbO concentrations of the MC at the RVCs during the late-task phase to a level close to that of the NRVCs. Unlike the PFC and MC, the HbO responses of the SSAC under the NRVCs remained higher than that of the RVCs after the visual cue appeared until it returned to the baseline level. The SSAC is mainly involved in spatial information processing and is responsible for the integration of visual and somatosensory information. We speculate that there is a lack of sufficient spatial cue information in NRVCs compared to RVCs, and thus more cognitive resources need to be mobilized to integrate spatial information for better motor planning.

### 4.2 β-values: cortical activation

From the activation maps based on β-values, cortical activation induced by RVCs was lower than that induced by NRVCs. General linear modeling (GLM) has been proved to be an effective means that can be used to examine hemodynamic responses in fNIRS analysis (Huppert, 2016; Santosa et al., 2020; Yucel et al., 2021). For example, a rapid event-related study with a randomized stimulus sequence demonstrated that a GLM-based modeling analysis method was able to detect event-related human brain activity in the occipital cortex from 4 to 9 s of fNIRS recordings (Plichta et al., 2007). In the current study, we considered β-values as the degree of cortical activation to quantify the correlation between brain regions and task conditions. We used β-values from two visual cue conditions to map the topography of cerebral activation by means of interpolation. The topographic maps depicted the hemodynamic responses elicited by the NRVCs and RVCs in each of the three task phases. In all three task phases, the SSAC hemodynamic responses under the influence of RVCs was smaller than that under the influence of NRVCs, especially in channels 11 and 13, whereas the differences were smaller for other ROIs or other channels. It may be due to the fact that our subjects were healthy adults whose automaticity and executive control were functioning normally compared to those with PD. Therefore, when subjects performed the task under RVCs or NRVCs, they may not need to expend additional cognitive resources in order to just complete a simple stepping task. But as mentioned before, the greatest difference between the two visual cues is that NRVCs lack more visuospatial elements than RVCs. In this case, even healthy individuals faced with this difference would have to struggle to mobilize enough cognitive resources to integrate visuospatial information in order to make more specific plans for gait. Besides that, the significant difference between the conditions exhibited by channel 8 in the early-task phase and in the late-task phase might suggest that stepping under NRVCs will take more effort. When examining the β-values over the entire time course, we found an overall decreasing trend. It may be due to the fact that as the task progressed, subjects gradually adapted to the stepping process and their gait became stable. At this point, the task conditions no longer served as stimuli, and the hemodynamic response they elicited decreased according to the stable or reduced neural activity.

### 4.3 Functional connectivity

Although both HbO concentration changes and cortical activation were lower under the RVC conditions, the strength of functional connectivity was stronger than that under the NRVC conditions at most stages. FC refers to the strong temporal correlation between spontaneous oscillations in brain regions with different functions (Fox and Raichle, 2007), reflecting important connections between different areas of the cerebral cortex. In the present study, FC values were calculated for all channel-channel pairs and were compared across three task phases as well as two visual cue conditions. The correlation matrix plotted by the FC values in each of the two visual cue conditions showed that the functional connectivity between channels within the PFC was stronger than other regions in the most phases and conditions, especially during the execution of the motor task. In the early-task phase, the MC internal channel pairs had a higher strength of functional connectivity under RVCs, while the SSAC internal channels had a stronger functional connectivity under both conditions. From a time-course perspective, the functional connectivity of the channel-channel pairs was the weakest in the pre-task phase, reached its strongest in the early-task phase, and decreased in the late-task phase compared to the early-task phase. Functional connectivity maps with significantly different FC values between channels in the two conditions were plotted based on the Table 1. The results showed that the number of channel-channel pairs with significant differences was the most in the early-task phase and that FC was stronger under RVCs than under NRVCs. It suggested that the most cognitive resources were recruited in the early-task phase, which may include delayed responses to hemodynamic activity elicited by motor preparation during the pre-task phase, as well as enhanced motor intention elicited by the RVC condition. Connections between channels under RVCs reflected enhanced connectivity in the frontoparietal network. The result was consistent with the findings from a recent brain functional network study that we mentioned earlier (Yan et al., 2023), which also found that brain networks under the RVCs had greater functional separation and functional integration. Although the HbO responses under RVCs were not higher than that under NRVCs in each ROI, the functional connectivity under RVCs was stronger than that under NRVCs, which exactly demonstrated the efficient allocation and utilization of cognitive resources under RVCs.

### 4.4 External cueing strategies for PD-FoG

These regularities will be used to explain the pattern of influence or mechanism of action of RVCs in alleviating FoG symptoms in PD patients. An EEG study of PD patients walking with rhythmic visual cues has shown that better gait under visual cues may be associated with changes in brain activity, especially in PD-FoG patients. The results of this study also suggested that PD patients require more cortical control when walking with visual cues (Stuart et al., 2021). For us, one of the reasons that RVCs can improve FoG symptoms may be that such visual cues can reduce the cognitive load of PD-FoG. Specifically, RVCs could provide visuospatial information to PD-FoG, so that they have enough cognitive resources to enhance executive control, thus compensating for deficits in automaticity. In line with a suggestion from a study on the role of the frontal lobes in complex walking in PD patients, this improves the efficiency of neural control in simple tasks, such as lower frontal activation (Maidan et al., 2016). On the other hand, in terms of the global efficiency of the brain, the RVCs can better regulate the allocation of cognitive resources and enhance the brain functional connectivity. However, all these speculations remain to be verified.

In addition to RVCs, rhythmic auditory cues (RACs) also have a certain improvement effect on PD-FoG. Perhaps different types of external cues have different mechanisms for FoG symptom improvement in PD patients, thus leading to different effects and aspects of improvement. The effects of RVCs and RACs on gait performance in PD-FoG have been compared in one study, which showed that both cues improved patients’ gait, but visual cues were more effective (Lee et al., 2012). It has also been shown that auditory cues improved gait in PD-FoG by decreasing gait cadence and freezing counts and increasing step length, but their effects on kinematic parameters were minimal. It has been suggested that external cues may reduce cognitive load by reducing the requirements for internal planning and preparation of movements (Rochester et al., 2007). There may be differences in the need for attentional resources between visual and auditory cues. Many people with PD-FoG are prone to triggering FoG during gait initiation, in which case RVCs have a more immediate effect than auditory cues (McCandless et al., 2016). When initiating gait with a visual cue, PD patients took a larger first step and experienced fewer lateral swings. RACs have been demonstrated to enhance PFC activation during walking in both young and old adults (Vitorio et al., 2018). This may be due to an age-independent increase in the executive control of the PFC in order to synchronize with the rhythm of the auditory cues during walking in subjects. In contrast to that study, our data indicated that PFC activation during stepping with RVCs was not greater in young adults than under NRVCs. The reason may be that visual cues provide more visuospatial information, which facilitates subjects to plan their gait and thus reduces the attentional resource consumption of PFC. It may also be due to the fact that the activation of the relevant cortex during stepping movements was much less than that of the actual walking process, which really required the mobilization of multiple resources to cooperate. In conclusion, both RVCs and RACs can improve gait in PD-FoG by affecting visuospatial information and kinematic parameters, but the mechanisms and effects of influence are different. Future studies may combine the advantages of various cues to provide better gait rehabilitation strategies for PD-FoG patients.

The present study has some limitations. We lacked PD patients with FoG symptoms to participate in this trial, and data from only healthy individuals might be less convincing. In terms of trial design, the walking motion during the trial was replaced with step-in-place because our fNIRS device does not have the advantage of being portable and wireless, and the length of the optical fiber limited the subjects’ range of motion. This may result in the inability to capture more complex dynamic activities like balance and proprioception. In addition to this, due to the small number of light poles, the brain regions covered are not accurate and it may happen that one channel covers more than one functional brain region, which may have an impact on the analysis of the results. These limitations need to be overcome one by one in future studies.

## 5 Conclusion

In the present study, we first investigated brain activity and functional connectivity between brain regions during the completion of stepping in healthy individuals under RVCs by fNIRS. In conclusion, the results suggest that RVCs not only reduces cognitive load, but also enhances functional connectivity between channels to maximize the benefits of cognitive resource allocation. In order to verify whether the findings of this study are applicable to PD patients, we are attempting to conduct further studies in PD-FoG patients.

## Data availability statement

The original data and materials presented in this article can be obtained from the corresponding authors upon request.

## Ethics statement

The studies involving humans were approved by the Ningbo Institute of Materials Technology and Engineering, Chinese Academy of Sciences. The studies were conducted in accordance with the local legislation and institutional requirements. The participants provided their written informed consent to participate in this study.

## Author contributions

JW: Conceptualization, Formal Analysis, Investigation, Methodology, Software, Writing – original draft. HZ: Conceptualization, Funding acquisition, Methodology, Software, Writing – review & editing. HC: Software, Writing – original draft. WJ: Data curation, Investigation, Writing – original draft. XW: Data curation, Writing – original draft. TM: Software, Writing – original draft. CW: Investigation, Writing – original draft. LL: Investigation, Writing – review & editing. YW: Investigation, Writing – review & editing. WF: Conceptualization, Supervision, Writing – review & editing. CS: Conceptualization, Funding acquisition, Methodology, Supervision, Writing – review & editing. GZ: Conceptualization, Funding acquisition, Methodology, Supervision, Writing – review & editing.
